# EphA3 Pay-Loaded Antibody Therapeutics for the Treatment of Glioblastoma

**DOI:** 10.3390/cancers10120519

**Published:** 2018-12-17

**Authors:** Carolin Offenhäuser, Fares Al-Ejeh, Simon Puttick, Kathleen S. Ensbey, Zara C. Bruce, Paul R. Jamieson, Fiona M. Smith, Brett W. Stringer, Benjamin Carrington, Adrian V. Fuchs, Craig A. Bell, Rosalind Jeffree, Stephen Rose, Kristofer J. Thurecht, Andrew W. Boyd, Bryan W. Day

**Affiliations:** 1 Department of Cell and Molecular Biology, QIMR Berghofer Medical Research Institute, Brisbane 4006, Australia; Carolin.Offenhauser@qimrberghofer.edu.au (C.O.); Fares.Al-Ejeh@qimrberghofer.edu.au (F.A.-E.); Kathleen.Ensbey@qimrberghofer.edu.au (K.S.E.); Zara.Bruce@qimrberghofer.edu.au (Z.C.B.); Jamieson.P@wehi.edu.au (P.R.J.); Fiona.Smith@qimrberghofer.edu.au (F.M.S.); Brett.W.Stringer@gmail.com (B.W.S.); Ben.Carrington@hansonwade.com (B.C.); Andrew.Boyd@qimrberghofer.edu.au (A.W.B.); 2 Australian Institute for Bioengineering and Nanotechnology, The University of Queensland, Brisbane 4072, Australia; S.Puttick@uq.edu.au (S.P.); adrianfuchs1@hotmail.com (A.V.F); C.Bell1@uq.edu.au (C.A.B.); K.Thurecht@uq.edu.au (K.J.T.); 3 Commonwealth Scientific and Industrial Research Organisation (CSIRO), Brisbane 4006, Australia; Stephen.Rose@csiro.au; 4 Centre for Advanced Imaging, The University of Queensland, Brisbane 4072, Australia; 5 Kenneth G. Jamieson Department of Neurosurgery, Royal Brisbane and Women’s Hospital, Brisbane 4029, Australia; Lindy.Jeffree@health.qld.gov.au; 6 School of Biomedical Sciences, Faculty of Health, Queensland University of Technology, Brisbane 4059, Australia

**Keywords:** EphA3, antibody drug conjugate, radioimmunotherapy, glioblastoma, stem cells

## Abstract

The EphA3 receptor has recently emerged as a functional tumour-specific therapeutic target in glioblastoma (GBM). EphA3 is significantly elevated in recurrent disease, is most highly expressed on glioma stem cells (GSCs), and has a functional role in maintaining self-renewal and tumourigenesis. An unlabelled EphA3-targeting therapeutic antibody is currently under clinical assessment in recurrent GBM patients. In this study, we assessed the efficacy of EphA3 antibody drug conjugate (ADC) and radioimmunotherapy (RIT) approaches using orthotopic animal xenograft models. Brain uptake studies, using positron emission tomography/computed tomography (PET/CT) imaging, show EphA3 antibodies are effectively delivered across the blood-tumour barrier and accumulate at the tumour site with no observed normal brain reactivity. A robust anti-tumour response, with no toxicity, was observed using EphA3, ADC, and RIT approaches, leading to a significant increase in overall survival. Our current research provides evidence that GBM patients may benefit from pay-loaded EphA3 antibody therapies.

## 1. Introduction

Glioblastoma (GBM) is the most common and aggressive adult malignant brain cancer. Despite surgical resection, radiation, and temozolomide chemotherapy, median survival is approximately 15 months [1,2]. Brain cancer sufferers have not seen meaningful increases in overall survival for decades. This is, in large part, due to the highly infiltrative and heterogeneous nature of these aggressive tumours [3]. GBM also displays complex cellular hierarchies containing self-renewing glioma stem cells (GSCs) with the ability to re-populate the tumour post-therapy.

Eph receptors are the largest family of receptor tyrosine kinases (RTKs). Whilst they have critical functions during embryonic development, they are typically expressed at low levels in normal adult tissues [4]. It is now established that numerous Eph receptors are re-expressed and functional in human cancers, making them attractive, relatively tumour-specific targets [5,6]. The ephrin type-A receptor 3 (EphA3) (formerly known as HEK) was first identified in 1992 as a surface antigen on pre-B lymphoblastic leukaemia cells [7]. Since then, EphA3 has been widely implicated in a number of developmental processes involving cell adhesion, cell migration, and tissue boundary formation. EphA3 has been shown to be elevated in a number of haematological cancers and solid tumours, with both oncogenic and tumour suppressive functions being described [8]. We recently reported that EphA3 has oncogenic functions in GBM [9,10]. Critically, we showed that EphA3 is expressed at low levels in a normal brain, but is most highly expressed on GSCs, where it has a functional role in survival and self-renewal. EphA3 knockdown (KD) induced tumour cell differentiation, apoptosis, and reduced tumour formation in vivo. More recently in GBM, EphA3 was shown to be significantly elevated in recurrent post-treatment versus primary treatment naïve disease. These findings further establish this receptor as an attractive tumour-specific target for brain cancer therapy [9,11,12]. Moreover, EphA3 has been shown to be over-expressed and functional on mesenchymal stromal cells in a number of human cancers, and EphA3 antibody targeting inhibited tumour growth by disrupting newly formed tumour microvasculature [13]. We previously generated the EphA3 monoclonal antibody (mAb) IIIA4 [7]. IIIA4 binds the EphA3 globular ephrin-binding domain with high affinity (Κ_D_ ~ 5 × 10^−10^ mol/L) with a low dissociation constant (Κ_D_ = 3 × 10^−4^/s) [14,15,16]. IIIA4, similar to how the high affinity ligand ephrin-A5, triggers rapid EphA3 activation and internalisation by inducing a conformation change allowing for the assembly of EphA3/ephrin-A5 signal clusters [17]. An investigator-sponsored Phase 0/1 clinical trial is currently underway using the EphA3-targeting monoclonal antibody (mAb) Ifabotuzumab [18] (Humanigen Inc., Brisbane, CA, USA), a humanised version of IIIA4, in patients with recurrent GBM. This study will confirm the safety and recommended dose to achieve optimal tumour penetration using positron emission tomography/computed tomography (PET/CT) imaging. This study is in part motivated by reports of the positive results of other antibody drug conjugate (ADC) therapy in the treatment of GBM. Depatuxizumab mafodotin (Depatux-m, formerly ABT-414) is an ADC against activated epidermal growth factor receptor (EGFR), conjugated, via a non-cleavable linker, to the cytotoxic microtubule-targeting agent monomethyl auristatin F (MMAF). Efficacy has been demonstrated as both a single agent and in combination with temozolomide (TMZ) [19,20].

EphA3 is predominantly expressed on the stem cell fraction in GBM and hence represents a logical target for further exploitation of immunotherapeutic approaches to this disease. To this end, we assessed the efficacy of EphA3 pay-loaded antibody strategies in GBM, using both EphA3 ADC and radioimmunotherapy (RIT) approaches in orthotopic GBM xenograft models. Our results shed light on further clinical evaluation of EphA3 targeting in GBM, and highlight this receptor as a tumour-specific candidate suitable for ADC or RIT strategies.

## 2. Results

### 2.1. The EphA3-Maytansine ADC Induces Potent Cell Killing In Vitro

**Figure 1:**
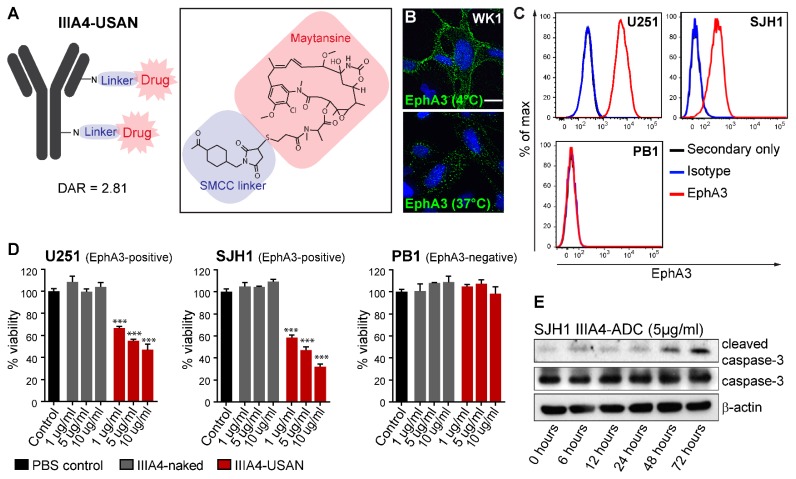
The EphA3-maytansine antibody drug conjugate (ADC) induces potent killing in vitro. (**A**) Schematic of IIIA4-USAN, an EphA3-specific monoclonal antibody (mAb) (IIIA4) conjugated to the tubulin-inhibitor maytansine (USAN) via a non-cleavable succinimidyl 4-(*N*-maleimidomethyl) cyclohexane-1-carboxylate (SMCC) linker. (**B**) Representative immunofluorescence images of EphA3-labelling with IIIA4 mAbs (shown in green) on the cell surface (top panel) and after antibody internalization (bottom panel) in the patient-derived glioblastoma (GBM) cell line WK1. (**C**) Flow cytometry analysis of EphA3 cell surface expression compared to isotype control in U251 and two primary GBM cell lines. (**D**) Cell viability of the EphA3^+^ U251 and SJH1 and EphA3^−^ PB1 GBM cell lines was measured by MTS colorimetric assay 7 days post IIIA4-USAN treatment. (**E**) SJH1 cells were treated with IIIA4-ADC (5 µg/mL) for the indicated times, followed by Western blot analysis. Scale bar represents 10 μm.

The IIIA4 antibody was deemed to be an excellent candidate for ADC therapy, as previous studies have shown that the IIIA4 antibody-EphA3 interaction induces rapid internalisation [17]. As a first step, we prepared an EphA3-ADC by conjugating the IIIA4 antibody to the cytotoxic microtubule-targeting agent maytansine (USAN), using a non-cleavable succinimidyl 4-(*N*-maleimidomethyl) cyclohexane-1-carboxylate (SMCC) linker, with a drug-to-antibody ratio (DAR) of 2.81 (Figure 1A and Figure S1A). A DAR of ~2–4 has been shown to be optimal for in vivo efficacy, as a low DAR reduces potency while a high DAR (~8) negatively affects antibody clearance and pharmacokinetics (PK) [21]. Bio-Layer Interferometry (BLI) Octet^®^, analysis showed that IIIA4 binding kinetics were unaffected following USAN conjugation (Figure S1B). Immunofluorescence (IF) analysis of EphA3-positive primary GBM cell lines, labelled with IIIA4 at 4 °C to prevent internalisation of receptor-antibody complexes, confirmed expression of the receptor on the cell membrane. To confirm receptor complexes were internalised following IIIA4 treatment, we performed IF labelling of EphA3 post-antibody treatment (IIIA4, 1 µg/mL at 37 °C for 20 min) (Figure 1B and Figure S1C). To assess IIIA4-USAN efficacy, we selected the EphA3-positive lines, U251 and SJH1, and an EphA3-negative line PB1. SJH1 and PB1 are primary GBM cultures, generated in-house (Q-Cell), and grown as glioma neural stem (GNS) cell cultures (Figure 1C) [22,23]. IIIA4-USAN showed a significant and specific reduction of cell viability in the EphA3-positive U251 and SJH1 lines 7 days post-treatment, which was accompanied with apoptotic cell death as judged by induction of caspase-3 cleavage (Figure 1D,E).

**Figure 2:**
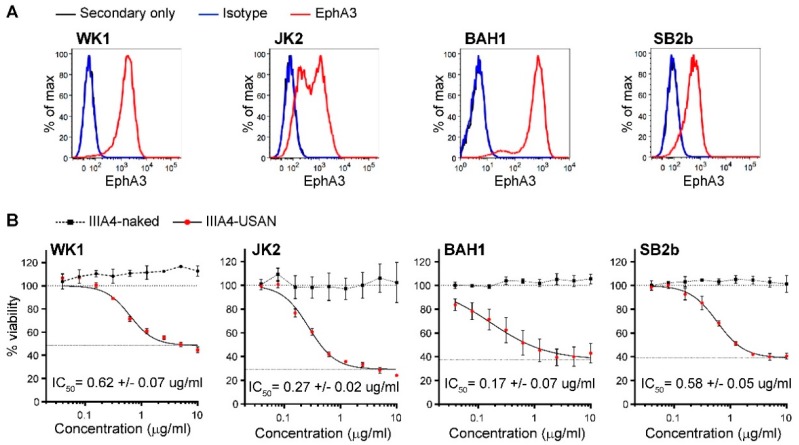
IC_50_ concentrations of IIIA4-USAN in primary models of GBM. (**A**) EphA3 cell surface flow cytometry profiles of four primary GBM cell lines. (**B**) Cell viability relative to the control was measured by MTS colorimetric assay and IC_50_ concentrations determined after 4 days of treatment with increasing doses of IIIA4-USAN versus IIIA4-naked.

We expanded our cytotoxicity studies to a panel of four Q-Cell EphA3-positive primary GBM GNS cultures, representing all GBM subtypes, classical (CL), proneural (PN) and mesenchymal (MES), (WK1-CL, JK2-PN, BAH1-PN, SB2b-MES) (Figure 2A) [24,25,26]. IIIA4-USAN was potent across these GBM cultures with a half maximal inhibitory concentration (IC_50_) of less than 0.7 µg/mL (Figure 2B).

### 2.2. IIIA4 Crosses the Blood-Tumour Barrier (BTB) and Accumulates Specifically in GBM

**Figure 3:**
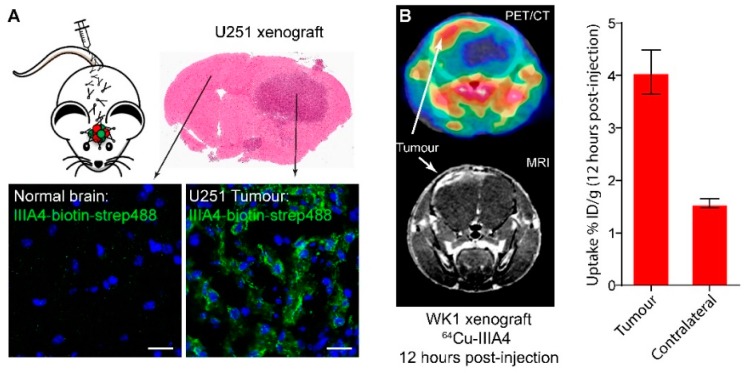
IIIA4 crosses the blood-tumour barrier (BTB) and accumulates specifically in GBM. (**A**) Tumour uptake of biotin-labelled IIIA4 (shown in green) 2 h post IV injection in a U251 orthotopic tumour xenograft, compared to adjacent normal brain was visualized with streptavidin-Alexa Fluor 488. (**B**) positron emission tomography/computed tomography (PET/CT) imaging and image quantification of ^64^Cu-labelled IIIA4 uptake into MRI-confirmed tumour tissue in an EphA3^+^ intracranial primary GBM tumour model (WK1). Images were acquired 12 h after IV injection of the labelled antibody. Scale bars represent 50 μm.

IIIA4 binds both mouse and human EphA3 with high affinity [17]. To assess IIIA4 brain penetration and tumour uptake, we engrafted the EphA3-positive U251 cell line into the right striatum of a NOD/SCID mouse and allowed the tumour to form orthotopically for 30 days. Biotin-labelled IIIA4 was then injected intravenously (IV) via the tail vein and allowed to circulate for two hours. The animal was then euthanised and coronal brain sections prepared, followed by IF analysis using streptavidin-Alexa Fluor 488. Results showed that the IIIA4-biotin accumulated specifically at the tumour site, while no staining was detected in surrounding normal brain (Figure 3A). To further assess tumour penetration, we engrafted the EphA3-positive primary model WK1 into the striatum of a NOD/SCID mouse. WK1 tumours were allowed to form for 60 days before PET/CT imaging was conducted 12 h post ^64^Cu-IIIA4 IV injection, followed by magnetic resonance imaging (MRI). PET/CT revealed specific tumour uptake with a minimal signal detected in contralateral normal brain (Figure 3B).

### 2.3. The EphA3-Maytansine ADC Induces A Potent GBM Anti-Tumour Response In Vivo

**Figure 4:**
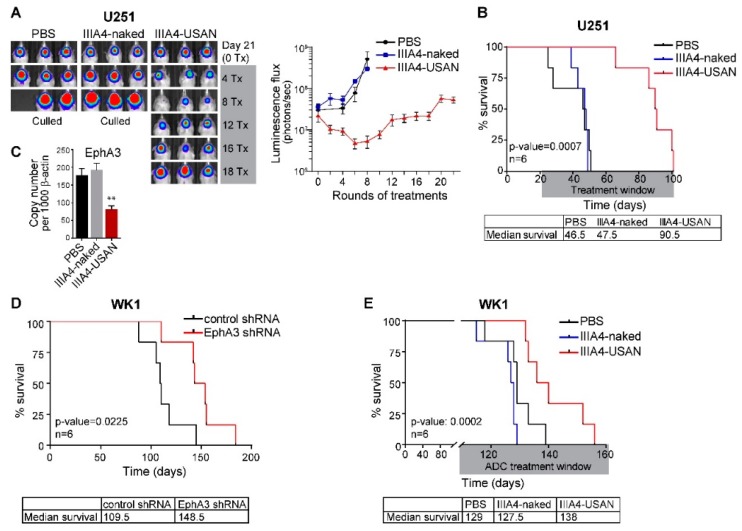
The EphA3-maytansine ADC induces a potent GBM anti-tumour response in vivo. (**A**) Luciferase-expressing U251 cells were engrafted into NRG mice. From day 21 after engraftment until endpoint, animals received twice weekly IV injections of IIIA4-USAN (10 mg/kg), IIIA4-naked (10 mg/kg), or PBS vehicle control. Representative images of tumour signals are shown. For complete analysis, see Figure S2A. (**B**) Kaplan-Meier survival analysis showing a significant increase in overall survival following IIIA4-USAN treatment. (**C**) qPCR analysis showing a significant (** *p* < 0.01) reduction in EphA3 mRNA levels in IIIA4-USAN-treated animals compared to controls. Tumors tissue from 4 animals per arm were analysed. (**D**) Kaplan Meier survival analysis showing a significant increase in overall survival of animals engrafted orthotopically with WK1 EphA3 shRNA-expressing cells compared to control shRNA-expressing cells. (**E**) Kaplan Meier survival analysis showing a significant increase in overall survival in NRG mice engrafted with the WK1 cells following IIIA4-USAN (10 mg/kg) treatment compared to controls.

To assess the efficacy of the IIIA4-USAN ADC, we first selected the EphA3-positive U251 model. Three weeks post-orthotopic injection, large tumours were detected via bioluminescent imaging. IIIA4-USAN therapy (10 mg/kg IV twice weekly) was commenced and compared to treatment with unlabelled IIIA4 (IIIA4-naked) or vehicle (PBS)-only arms. No signs of toxicity were observed at any time during the course of the up to 22 IIIA4-USAN injections. Weekly imaging revealed an initial anti-tumour response, which appeared to reduce tumour burden to near completion in some animals after eight treatments (Figure 4A and Figure S2A). Tumours rapidly progressed in both control arms and all control animals had been euthanised five weeks after treatment initiation from advanced tumour burden. Despite continuation of IIIA4-USAN injections, tumours recurred and progressed in all animals. IIIA4-USAN led to a significant (*p* = 0.0007, *n* = 6) increase in overall survival compared to controls. Notably, IIIA4-USAN almost doubled overall survival. Median survival in days was 46.5 for PBS and 47.5 for IIIA4-naked, versus 90.5 days for IIIA4-USAN (Figure 4B). Post mortem, we examined EphA3 mRNA levels in controls versus treated animals by qPCR. Tumours were collected from four animals per group and expression analysed. IIIA4-USAN treatment led to a significant (*p* < 0.01) reduction in EphA3 mRNA levels in these tumours (Figure 4C). We repeated our initial U251 IIIA4-USAN animal study, on this occasion we allowed tumours to form for six weeks before treatment was commenced. Weekly imaging revealed a similar initial robust anti-tumour response, while tumours rapidly progressed in both control arms. ADC treatment significantly increased overall survival (*p* = 0.0024, *n* = 5). Median survival in days was 56 for PBS and 60 for IIIA4-naked, versus 92 days for IIIA4-USAN (Figure S2B). We have previously shown that shRNA-mediated knockdown (KD) of EphA3 in U251 cells prolongs survival following orthotopic engraftment [9]. To confirm that EphA3 has a similar oncogenic role in the WK1 primary model, we conducted a comparable orthotopic animal study. WK1 control shRNA cells were compared to constitutive EphA3 shRNA expressing cells. Effective EphA3 KD in WK1 was confirmed as part of a previous study [9]. EphA3 KD led to a significant increase in overall survival (*p* = 0.0225, *n* = 6). Median survival in days was control 109.5 for shRNA versus 148.5 for EphA3 KD (Figure 4D). We next assessed IIIA4-USAN, using the WK1 model as a single agent (10 mg/kg IV twice weekly). A significant increase (*p* = 0.0002, *n* = 6) in overall survival was observed from the IIIA4-USAN compared to PBS and IIIA4-naked (Figure 4E).

### 2.4. EphA3-Targeting RIT Induces A GBM Anti-Tumour Response In Vivo

**Figure 5:**
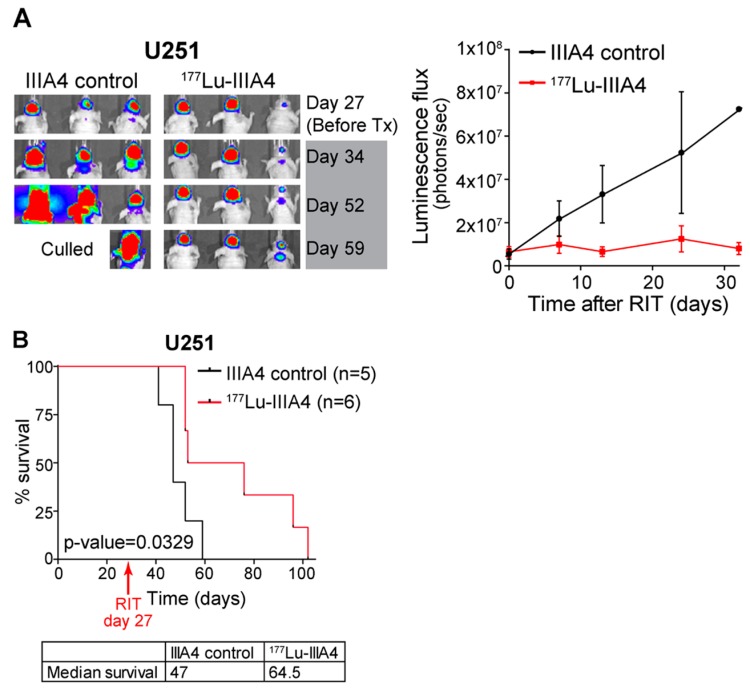
EphA3-targeting RIT induces a GBM anti-tumour response in vivo. (**A**) Luciferase-expressing U251 cells were engrafted orthotopically and, on day 27, animals received a single dose of 450 MBq/kg dose of IIIA4-DOTA-^177^Lu (^177^Lu-IIIA4) or IIIA4-DOTA control (IIIA4 control). Representative images of tumour signals are shown. (**B**) Kaplan-Meier survival analysis showing a significant increase in overall survival following a single dose of ^177^Lu-IIIA4 (450 MBq/kg) treatment.

To examine if EphA3-targeting RIT could have benefit as a GBM therapy we radiolabelled IIIA4-DOTA with the β particle-emitting radionuclide lutetium-177 (^177^Lu-IIIA4). Established U251 xenografts in BALB/c nude mice were treated with a single dose of ^177^Lu-IIIA4 (450 MBq/kg, equivalent to ~9 MBq per animal), administered IV. No signs of toxicity were observed at any time during the course of the experiment. Compared to the unlabelled IIIA4-DOTA control, a significant anti-tumour response and a delayed onset of tumour progression, up to 32 days, was detected by imaging (Figure 5A). This delay is significant considering the 6.7 days half-life of ^177^Lu, and that by day 32 approximately five half-lives have passed. Tumours rapidly progressed in the control arm and all control animals had been euthanised 32 days after commencing treatment, due to advanced tumour burden. ^177^Lu-IIIA4 led to a significant (*p* = 0.0329) increase in overall survival compared to the control arm. Median survival in days was 47 for IIIA4-DOTA-control versus 64.5 for ^177^Lu-IIIA4 (Figure 5B).

## 3. Discussion

Since 2005, when TMZ was adopted into the clinical management of GBM, no therapy has significantly improved overall patient survival, prompting a search for alternative therapeutic modalities [1,2]. ADC therapy has emerged as an effective part of anti-cancer treatment. A recent example is the HER2 mAb Trastuzumab coupled to the maytansinoid anti-microtubule agent (DM1) for the treatment of HER2-positive breast cancer [27,28,29]. In GBM, Depatux-m (ABT-414), an ADC against activated EGFR is showing promise as an effective therapy [19,20]. A large body of data on the role of EphA3 biology in cancer has identified this receptor as one of the most promising targets for therapy, based on low expression levels in normal adult tissues and the tumour-promoting properties, including stem cell maintenance, angiogenesis, metastatic functions, and enriched expression in recurrent disease [30]. Here, we build upon our existing body of work and the work of others to characterize EphA3 as a tumour-specific therapeutic target for the treatment of GBM [9,10,11,12]. Our findings show that EphA3 is an effective target for antibody-based pay-loaded therapy in GBM. PET/CT imaging showed that the IIIA4 antibody readily enters the tumour in our orthotopic models, accumulating specifically at the tumour site with minimal uptake in normal brain. Importantly, we observed no toxicity from either EphA3 ADC or RIT treatment. IIIA4 has equal affinity for both mouse and human EphA3, allowing accurate assessment of off-target toxicity in our animal studies. EphA3 ADC or RIT were both very well tolerated, in the case of one animal study, up to 22 doses of IIIA4-USAN were administered over an eleven-week period with no toxic response. In all our animal studies, therapy was commenced only after large tumours had already formed in the brain. Despite this, both ADC and RIT therapies were able to significantly reduce tumour burden. In one experiment, although ADC therapy was not commenced until six weeks post engraftment, efficacy was still achieved, leading to a significant increase in overall survival in the treated group. Thus, both pay-loading strategies were shown to be effective, whereas the IIIA4 antibody alone showed no effect on tumour progression. The EphA3-targeting mAb Ifabotuzumab, a humanised version of IIIA4, has been trialed in its naked form in patients with advanced haematologic malignancies [18]. Results from this study found that the antibody was well-tolerated. While some clinical responses were observed, overall responses were modest. An investigator-sponsored Phase 0/1 clinical trial has been instigated to assess Ifabotuzumab in patients with recurrent GBM (NCT03374943). This study will confirm safety in a solid tumour setting and determine the biodistribution and pharmacokinetics of ^89^Zr-Ifabotuzumab using PET/CT imaging. In addition, PET/CT imaging could be used to screen EphA3 levels in patient tumours prior to commencing Ifabotuzumab therapy. Though efficacy of naked Ifabotuzumab is yet to be determined in patients with GBM, it might be expected that greater responses could be achieved using pay-loaded approaches in these aggressive diseases. With this in mind, we chose to assess EphA3 targeting antibody pay-loaded strategies in pre-clinical GBM models to inform potential future trial design.

Ultimately, all tumours recurred using both pay-loaded strategies. This was despite ADC therapy being administered throughout the course of the experiments. In seeking to understand the escape from the initial response, it was notable that ADC therapy also significantly reduced EphA3 receptor levels in recurrent tumours, indicating that escape might be partly mediated by down-regulation of EphA3. Alternatively, this finding could indicate that therapy was specifically targeting the EphA3 positive tumour compartment, reducing overall receptor levels. In recent years, acquired resistance to ADCs has emerged as a challenge for this class of cancer therapeutics. Most patients treated with Trastuzumab-maytansine (T-DM1), while initially responsive, develop acquired drug resistance to this therapy [31]. Preclinical studies have recently revealed several possible resistance mechanisms, including decreased cell surface antigen expression, altered intracellular trafficking, impaired lysosomal degradation, and payload removal via multiple drug resistance transporters [32]. This highlights the need for drugs which target multiple aspects of tumour biology in order to develop effective combinations of therapies, rather than single agents alone. However, despite these limitations, our pre-clinical study indicates that GBM sufferers may benefit from pay-loaded EphA3 antibody targeting strategies and supports further clinical evaluation of these therapies.

## 4. Materials and Methods

### 4.1. Primary Cell Cultures

We have developed a characterised GBM patient-derived cell line resource (Q-Cell) [23], in which GBM lines are maintained as glioma neural stem cell (GNS) cultures [22] or as neurosphere cultures using StemPro NSC SFM (Invitrogen, Carlsbad, CA, USA) as per manufacturer’s guidelines. Characterisation data is freely available from: https://www.qimrberghofer.edu.au/q-cell/. U251-MG GBM cells were obtained from the ATCC (Manassas, VA, USA) and cultured as GNS cultures as described above. Luciferase-expressing lines were generated by transducing U-251-MG and WK1 cells with Firefly Luciferase Lentifect™ Purified Lentiviral Particles (GeneCopoeia, Rockville, MD, USA). 70–80% confluent cells were transduced with 2 µL of Lentifect particles in 2 mL of culture medium, supplemented with 8 µg/mL polybrene per well of a 6-well plate. After adding the virus, cells were centrifuged at 500× *g* in a swing-out rotor for 45 min at room temperature and subsequently cultured for 24 h before the medium was replaced. Puromycin was added 72 h after transduction to select successfully transduced cells.

### 4.2. ADC Preparation

IIIA4 was purified in-house as previously described [17]. To conjugate IIIA to the non-cleavable linker SMCC, sulfo-SMCC (2.2 mg in 220 µL DMSO) was added dropwise to IIIA4 in PBS (8 mL at 9.17 mg/mL) at 4 °C and stirred overnight. IIIA4-SMCC was purified by centrifugal filtration (Amicon Ultra-15 centrifugal filters, 3 kDa MWCO). The SMCC linker to antibody ratio of 7.2 SMCC units per IIIA4 was determined by Matrix Assisted Laser Desorption Ionisation-Time of Flight Mass Spectroscopy (MALDI-ToF MS). Maytansine DM1 (1.62 mg in 162 µL DMSO, Abcam, Cambridge, UK) was added dropwise to IIIA4-SMCC (8.2 mL at 9.26 mg/mL) in PBS at 4 °C, stirred overnight and purified by centrifugal filtration (Amicon Ultra-15 centrifugal filters, 3 kDa MWCO). A drug-to-antibody (DAR) ratio of 2.81 USAN units per IIIA4-SMCC was determined by MALDI-ToF. MALDI-ToF MS was conducted on a Bruker Autoflex III equipped with a nitrogen laser delivering 3 ns laser pulses at 337 nm. A matrix solution was prepared consisting of sinapinic acid (saturated) in MeCN containing 0.1% *v*/*v* trifluoroacetic acid. The antibody solutions (in PBS) were diluted 10× with Milli-Q water, then mixed in an Eppendorf tube with the matrix solution in a 1:1 ratio. The resulting solutions were then applied to a polished steel target (1 µL) followed be solvent evaporation to prepare a thin matrix/analyte film. Samples were analysed in positive linear mode over a mass range of 20–200 kDa with an average of 10,000 shots. The average number of maleimide reactive groups per IIIA4-SMCC for USAN attachment was calculated from the mass difference between IIIA4-SMCC and IIIA4 divided by MW_SMCC_ after conjugation (220.1 Da). Subsequently, the DAR was calculated from the mass difference between IIIA4-USAN and IIIA4-SMCC divided by MW_USAN_ after conjugation (738.3 Da).

### 4.3. Bio-Layer Interferometry (BLI)

Antibody binding kinetics of IIIA4-naked and IIIA4-USAN to EphA3-Fc-loaded biosensors was compared by Bio-Layer Interferometry using the Octet^®^ RED system (ForteBio, Fremont, CA, USA). EphA3-Fc protein (in-house) was conjugated to biotin using a 1:1 molar ratio of protein to EZ-Link Sulfo NHS-LC-LC-Biotin (ThermoFisher Scientific, Waltham, MA, USA) following the manufacturer’s instructions. Excess biotin was removed using Zeba Spin desalting columns 7K MWCO (ThermoFisher Scientific). Biotinylated EphA3-Fc protein (50 µg/mL) was loaded onto streptavidin biosensors (ForteBio) as per the manufacturer’s instructions, and binding of IIIA4-naked and IIIA4-USAN (30 µg/mL) to individual sensors recorded. On reaching saturation, the probes were exchanged into 1× kinetics buffer (1 mM Phosphate, 15 mM NaCl, 0.005% Tween 20 and 0.1 mg/mL BSA) to allow dissociation.

### 4.4. Cell Viability Assay

To assess cell viability, 5 × 10^3^ cells per well were seeded in 96-well plates in GNS conditions and incubated overnight. Cells were incubated with IIIA4-naked and IIIA4-USAN at the indicated concentrations or medium alone (control) for seven days before cell viability was assessed by MTS assay as per manufacturer’s instructions (CellTiter 96 Aqueous One Cell Proliferation Assay; Promega, Madison, WI, USA). To measure dose response curves, cells were incubated with nine 1:2 serial dilutions, starting from 10 µg/mL, for 96 h before cell viability was assessed. Responses were normalised to percent viability of control and maximal effect and half-maximal inhibitory concentration (IC_50_) determined by four-parameter nonlinear regressing analysis (top plateau constrained to 100%) using GraphPad Prism 7 software (GraphPad, San Diego, CA, USA).

### 4.5. Caspase-3 Activation

To assess caspase activation, cells were cultured in GNS conditions and treated with 5 ug/mL IIIA4-ADC for the indicated times, or incubated in medium alone. Cells were lysed and protein expression of cleaved caspase-3 (Cell Signaling Technology, Danvers, MA, USA, #9661, 1:1000), total caspase-3 (Cell Signaling Technology, #9662, 1:1000) and β-actin (Cell Signaling Technology, #9700, 1:5000) determined by Western blot analysis as previously described [33].

### 4.6. Immunofluorescence Analysis

Cells were seeded on coverslips and cultured overnight in GNS conditions. Cells were incubated with IIIA4 antibody at 4 °C (to prevent internalisation of antibody receptor complexes) or 37 °C for 20 min. Cells were washed twice in PBS and fixed in 4% paraformaldehyde in PBS for 30 min at room temperature. Cells were permeabilised with 0.25% Triton X-100 in PBS for 10 min at room temperature and EphA3 receptor-antibody complexes visualised with Alexa Fluor 488-conjugated secondary antibodies (ThermoFisher Scientific). Nuclei were labelled with DAPI. For in vivo uptake of IIIA4 antibody into intracranially engrafted tumours, a U251-MG tumour-bearing mouse was injected with a single dose of biotin-conjugated IIIA4 antibody (10 mg/kg). The antibody was allowed to circulate for 2 h before the mouse was euthanised and the brain removed and fixed in 10% neutral buffered formalin. IIIA4 antibody uptake was visualised in coronal cross-sections using Alexa Fluor 488 streptavidin (ThermoFisher Scientific), and nuclei were labelled with DAPI.

### 4.7. Flow Cytometry

Cell surface expression of EphA3 was analysed using the IIIA4 antibody (in-house, 1:100). IgG1 isotype control antibody (Abcam, 1:100) was used to indicate background staining. Cells were washed twice in ice-cold PBS and blocked for 20 min in 1% bovine serum albumin (BSA) in PBS on ice. After blocking, cells were incubated with primary antibodies for 20 min on ice, washed and incubated with secondary Alexa Fluor 488-conjugated antibodies for 20 min on ice in the dark (ThermoFisher Scientific). EphA3 expression levels were examined using a LSR Fortessa flow cytometer (BD, Franklin Lakes, NJ, USA) and acquired data analysed using FlowJo (FlowJo, LLC, CA, USA) software.

### 4.8. Animal Studies

All mice experiments were performed according to the National Health and Medical Research Council (2013) Australian code for the care and use of animals for scientific purposes, under experimental protocols approved by the QIMR Berghofer Animal Ethics Committee, P1572-A1412-613M approved 17 December 2014; P1173-A0812-608M approved 12 December 2008. For intracranial (orthotopic) xenograft studies, five week-old female NOD/SCID (NOD.CB17-Prkdc scid/Arc), BALB/c Nude (BALB/c-Foxn1^nu^/Arc) or NRG mice (NOD.Cg-Rag1^tm1MOM^ IL2rg^tm1Wjl/SzJ^) animals were sourced from the Animal Resources Centre (ARC) in Canning Vale, Western Australia). Mice were anaesthetised using 2% isoflurane in oxygen and engrafted intracranially using a small animal stereotactic device. Mice were given analgesia (Meloxicam (Ilium) 5 mg/kg, delivered subcutaneously) 30 min prior to surgery and again the following day. Tumour growth was monitored weekly by bioluminescence imaging. To generate bioluminescence signals, mice were anaesthetised with 2% isoflurane in oxygen, injected IP with 100 µL of 5 mg/mL D-Luciferin (Pure Science Ltd, Porirua, New Zealand) in PBS and imaged using the IVIS Spectrum in vivo imaging system (Perkin Elmer, Waltham, MA, USA). Following tumour formation, mice were randomised and tumour-matched based on bioluminescence imaging results. A non-specific antibody (1A7, in-house, 500 μg/mouse) was injected into the peritoneum 2 h prior to ADC therapy to block unspecific tissue uptake by unoccupied Fc receptors in immune-compromised mice. IIIA4-naked and IIIA4-USAN in PBS were administered intravenously at 10mg/kg twice weekly via the lateral tail vain. Mice were monitored daily for signs of illness or tumour burden as per our ethical guidelines, animal monitoring criteria, and scoring. At the endpoint, animals were euthanised by cervical dislocation.

### 4.9. Imaging

#### 4.9.1. Generation of IIIA4-NOTA

IIIA4 (1 mg) was buffer exchanged into 0.1 M Na_2_CO_3_ (pH 9.2) and pSCN-Bn-NOTA (35 μg in 3.5 μL DMSO, Macrocyclics) added. The reaction mixture was incubated for 1 h at 37 °C with gentle agitation followed by purification by size exclusion chromatography (ÄKTA PrimePlus fitted with a 5 mL, HiTrap Desalting G25 column running a mobile phase of 0.1 M PBS at 0.5 mL/min, GE Life Sciences, NSW, Australia).

#### 4.9.2. Radiolabelling

IIIA4-NOTA (200 μg) was buffer exchanged into 0.1 M NH_3_OAc (pH 5.5) and incubated with 100 MBq ^64^CuOAc for 1 h at 37 °C with gentle agitation followed by purification by centrifugal filtration (Zeba Spin Desalting Column, 7K MWCO, 0.5 mL equilibrated with 1× PBS, ThermoFisher). Radiochemical purity was assessed by iTLC (50 mM EDTA mobile phase using silica-gel impregnated glass microfiber paper strips (iTLC-SG, Agilent, Santa Clara, CA, USA). Radiochemical purity was >98%, radiochemical yield was 70% and specific activity was 350 MBq/mg.

#### 4.9.3. PET/CT Imaging

NOD/SCID (*n* = 3) mice bearing intracranial WK1 tumours were injected with IIIA4-NOTA-^64^Cu (5–7 MBq) via the lateral tail vein. At 12 h post injection, mice were anaesthetised and maintained using 2% isoflurane in oxygen at a flow rate of 2 L/min and positioned on the scanning bed of an Inveon Preclinical PET/CT system (Siemens, Munich, Germany). A 30 min PET image was acquired followed by a CT for anatomical registration and attenuation correction. PET images were reconstructed using the OSEM-2D reconstruction algorithm in Inveon Acquisition Workspace (IAW, Siemens) correcting for isotope half-life, attenuation, and ^64^Cu detector efficiency.

#### 4.9.4. Magnetic Resonance Imaging (MRI)

MRI images were acquired 24 h following PET/CT imaging. Mice were anaesthetised and maintained using 2% isoflurane in oxygen at a flow rate of 2 L/min. Immediately prior to acquisition of the MRI image, mice were injected with 50 μL of a 0.2 M solution of Magnevist (Bayer, Leverkusen, Germany) in 1× PBS. Mice were placed on the scanning bed of a Brüker 7T ClinScan (Brüker, Billerica, MA, USA) fitted with a 23 mm volume coil and 3D T_1_ weighted contrast enhanced images were acquired using a VIBE sequence (TR = 12 ms, TE = 1.78 ms, FA = 21°, 0.12 mm isotropic resolution).

#### 4.9.5. Image Analysis

Decay-corrected PET images and contrast-enhanced MRI images were co-registered to the CT image space using Inveon Research Workspace (Siemens). The brain was segmented from the CT image and applied as a mask to the PET and MRI images. Contrast-enhancing tumour was segmented from the MRI image by thresholding the brain volume voxel histogram and the segmented volume duplicated and horizontally reflected to form tumour and contralateral brain volumes of interest (VOIs). These VOIs were transformed onto the co-registered PET image and mean %ID/g within each VOI was measured.

### 4.10. Radioimmunotherapy (RIT)

The EphA3 mAb IIIA4 was conjugated to DOTA-NHS (Macrocyclics, Plano, TX, USA), radiolabelled with ^177^LuCl_3_, and purified using 100 kDA-cutoff microconcentrators (Millipore, Burlington, MA, USA) as previously described [9,34,35]. The specific activity of ^177^Lu-IIIA4 ranged from 225 and 260 MBq/mg, and radiolabelling efficiency was >98% as judged by standard, instant iTLC-SG [34]. Mice were injected intravenously with ^177^Lu-IIIA4 once only at 450 MBq/kg dose (equivalent to 1.7–2.0 mg/kg of IIIA4 mAb dose).

### 4.11. Statistical Analysis

In vivo experiments—sample size or replicate number (designated as “*n*”)—for each experiment is indicated in the figure. Charts and survival plots were generated using GraphPad Prism 7 software. A log-rank (Mantel-Cox) test was used to determine the significance between experimental groups. *p-*values are as indicated and * *p* ≤ 0.05 was considered to be statistically significant. In vitro experiments—a Student’s *t*-test determined the probability of difference—* *p* < 0.05 was considered significant; all statistical tests were two-sided.

## 5. Conclusions

Our data demonstrate that EphA3 can be effectively targeted using both ADC or RIT approaches, and that EphA3 mAbs cross the BTB to allow tumour-specific targeting. Our pre-clinical study supports further clinical evaluation of pay-loaded EphA3 antibody therapies in patients with GBM.

## Supplementary Materials

The following are available online at http://www.mdpi.com/2072-6694/10/12/519/s1, Figure S1: Validation of the EphA3-maytansine ADC IIIA4-USAN, Figure S2: In vivo efficacy of the EphA3-maytansine ADC IIIA4-USAN.

## References

[1] Stupp R., Mason W.P., van den Bent M.J., Weller M., Fisher B., Taphoorn M.J., Belanger K., Brandes A.A., Marosi C., Bogdahn U. (2005). Radiotherapy plus concomitant and adjuvant temozolomide for glioblastoma. N. Engl. J. Med..

[2] Stupp R., Hegi M.E., Mason W.P., van den Bent M.J., Taphoorn M.J., Janzer R.C., Ludwin S.K., Allgeier A., Fisher B., Belanger K. (2009). Effects of radiotherapy with concomitant and adjuvant temozolomide versus radiotherapy alone on survival in glioblastoma in a randomised phase III study: 5-year analysis of the EORTC-NCIC trial. Lancet Oncol..

[3] Hanahan D., Weinberg R.A. (2011). Hallmarks of cancer: The next generation. Cell.

[4] Pasquale E.B. (2005). Eph receptor signalling casts a wide net on cell behaviour. Nat. Rev. Mol. Cell Biol..

[5] Pasquale E.B. (2010). Eph receptors and ephrins in cancer: Bidirectional signalling and beyond. Nat. Rev. Cancer.

[6] Boyd A.W., Bartlett P.F., Lackmann M. (2013). Therapeutic targeting of EPH receptors and their ligands. Nat. Rev. Drug Discov..

[7] Boyd A.W., Ward L.D., Wicks I.P., Simpson R.J., Salvaris E., Wilks A., Welch K., Loudovaris M., Rockman S., Busmanis I. (1992). Isolation and characterization of a novel receptor-type protein tyrosine kinase (hek) from a human pre-B cell line. J. Biol. Chem..

[8] Pasquale E.B. (2008). Eph-ephrin bidirectional signaling in physiology and disease. Cell.

[9] Day B.W., Stringer B.W., Al-Ejeh F., Ting M.J., Wilson J., Ensbey K.S., Jamieson P.R., Bruce Z.C., Lim Y.C., Offenhauser C. (2013). EphA3 maintains tumorigenicity and is a therapeutic target in glioblastoma multiforme. Cancer Cell.

[10] Day B.W., Stringer B.W., Boyd A.W. (2014). Eph receptors as therapeutic targets in glioblastoma. Br. J. Cancer.

[11] Qazi M.A., Vora P., Venugopal C., Adams J., Singh M., Hu A., Gorelik M., Subapanditha M.K., Savage N., Yang J. (2018). Cotargeting Ephrin Receptor Tyrosine Kinases A2 and A3 in Cancer Stem Cells Reduces Growth of Recurrent Glioblastoma. Cancer Res..

[12] Ferluga S., Tome C.M., Herpai D.M., D’Agostino R., Debinski W. (2016). Simultaneous targeting of Eph receptors in glioblastoma. Oncotarget.

[13] Vail M.E., Murone C., Tan A., Hii L., Abebe D., Janes P.W., Lee F.T., Baer M., Palath V., Bebbington C. (2014). Targeting EphA3 Inhibits Cancer Growth by Disrupting the Tumor Stromal Microenvironment. Cancer Res..

[14] Lackmann M., Mann R.J., Kravets L., Smith F.M., Bucci T.A., Maxwell K.F., Howlett G.J., Olsson J.E., Vanden Bos T., Cerretti D.P. (1997). Ligand for EPH-related kinase (LERK) 7 is the preferred high affinity ligand for the HEK receptor. J. Biol. Chem..

[15] Lackmann M. (2001). Isolation and characterization of “orphan-RTK” ligands using an integrated biosensor approach. Methods Mol. Biol..

[16] Smith F.M., Vearing C., Lackmann M., Treutlein H., Himanen J., Chen K., Saul A., Nikolov D., Boyd A.W. (2004). Dissecting the EphA3/Ephrin-A5 interactions using a novel functional mutagenesis screen. J. Biol. Chem..

[17] Vearing C., Lee F.T., Wimmer-Kleikamp S., Spirkoska V., To C., Stylianou C., Spanevello M., Brechbiel M., Boyd A.W., Scott A.M. (2005). Concurrent binding of anti-EphA3 antibody and ephrin-A5 amplifies EphA3 signaling and downstream responses: Potential as EphA3-specific tumor-targeting reagents. Cancer Res..

[18] Swords R.T., Greenberg P.L., Wei A.H., Durrant S., Advani A.S., Hertzberg M.S., Jonas B.A., Lewis I.D., Rivera G., Gratzinger D. (2016). KB004, a first in class monoclonal antibody targeting the receptor tyrosine kinase EphA3, in patients with advanced hematologic malignancies: Results from a phase 1 study. Leuk. Res..

[19] Gan H.K., Reardon D.A., Lassman A.B., Merrell R., van den Bent M., Butowski N., Lwin Z., Wheeler H., Fichtel L., Scott A.M. (2018). Safety, pharmacokinetics, and antitumor response of depatuxizumab mafodotin as monotherapy or in combination with temozolomide in patients with glioblastoma. Neuro Oncol..

[20] Lassman A.B., van den Bent M.J., Gan H.K., Reardon D.A., Kumthekar P., Butowski N., Lwin Z., Mikkelsen T., Nabors L.B., Papadopoulos K.P. (2018). Safety and efficacy of depatuxizumab mafodotin + temozolomide in patients with EGFR-amplified, recurrent glioblastoma: Results from an international phase I multicenter trial. Neuro Oncol..

[21] Hamblett K.J., Senter P.D., Chace D.F., Sun M.M., Lenox J., Cerveny C.G., Kissler K.M., Bernhardt S.X., Kopcha A.K., Zabinski R.F. (2004). Effects of drug loading on the antitumor activity of a monoclonal antibody drug conjugate. Clin. Cancer Res..

[22] Pollard S.M., Yoshikawa K., Clarke I.D., Danovi D., Stricker S., Russell R., Bayani J., Head R., Lee M., Bernstein M. (2009). Glioma stem cell lines expanded in adherent culture have tumor-specific phenotypes and are suitable for chemical and genetic screens. Cell Stem Cell.

[23] Day B.W., Stringer B.W., Wilson J., Jeffree R.L., Jamieson P.J., Ensbey K.S., Bruce Z.C., Inglis P., Allan S., Winter C. (2013). Glioma Surgical Aspirate: A Viable Source of Tumor Tissue for Experimental Research. Cancers.

[24] Verhaak R.G., Hoadley K.A., Purdom E., Wang V., Qi Y., Wilkerson M.D., Miller C.R., Ding L., Golub T., Mesirov J.P. (2010). Integrated genomic analysis identifies clinically relevant subtypes of glioblastoma characterized by abnormalities in PDGFRA, IDH1, EGFR, and NF1. Cancer Cell.

[25] Brennan C., Momota H., Hambardzumyan D., Ozawa T., Tandon A., Pedraza A., Holland E. (2009). Glioblastoma subclasses can be defined by activity among signal transduction pathways and associated genomic alterations. PLoS ONE.

[26] Wang Q., Hu B., Hu X., Kim H., Squatrito M., Scarpace L., de Carvalho A.C., Lyu S., Li P., Li Y. (2017). Tumor Evolution of Glioma-Intrinsic Gene Expression Subtypes Associates with Immunological Changes in the Microenvironment. Cancer Cell.

[27] Hudis C.A. (2007). Trastuzumab—Mechanism of action and use in clinical practice. N. Engl. J. Med..

[28] Burris H.A., Rugo H.S., Vukelja S.J., Vogel C.L., Borson R.A., Limentani S., Tan-Chiu E., Krop I.E., Michaelson R.A., Girish S. (2011). Phase II study of the antibody drug conjugate trastuzumab-DM1 for the treatment of human epidermal growth factor receptor 2 (HER2)-positive breast cancer after prior HER2-directed therapy. J. Clin. Oncol..

[29] Krop I.E., Beeram M., Modi S., Jones S.F., Holden S.N., Yu W., Girish S., Tibbitts J., Yi J.H., Sliwkowski M.X. (2010). Phase I study of trastuzumab-DM1, an HER2 antibody-drug conjugate, given every 3 weeks to patients with HER2-positive metastatic breast cancer. J. Clin. Oncol..

[30] Janes P.W., Slape C.I., Farnsworth R.H., Atapattu L., Scott A.M., Vail M.E. (2014). EphA3 biology and cancer. Growth Factors.

[31] Barok M., Joensuu H., Isola J. (2014). Trastuzumab emtansine: Mechanisms of action and drug resistance. Breast Cancer Res..

[32] Loganzo F., Sung M., Gerber H.P. (2016). Mechanisms of Resistance to Antibody-Drug Conjugates. Mol. Cancer Ther..

[33] Day B.W., Stringer B.W., Spanevello M.D., Charmsaz S., Jamieson P.R., Ensbey K.S., Carter J.C., Cox J.M., Ellis V.J., Brown C.L. (2011). ELK4 neutralization sensitizes glioblastoma to apoptosis through downregulation of the anti-apoptotic protein Mcl-1. Neuro Oncol..

[34] Al-Ejeh F., Shi W., Miranda M., Simpson P.T., Vargas A.C., Song S., Wiegmans A.P., Swarbrick A., Welm A.L., Brown M.P. (2013). Treatment of triple-negative breast cancer using anti-EGFR-directed radioimmunotherapy combined with radiosensitizing chemotherapy and PARP inhibitor. J. Nucl. Med. Off. Publ. Soc. Nucl. Med..

[35] Al-Ejeh F., Pajic M., Shi W., Kalimutho M., Miranda M., Nagrial A.M., Chou A., Biankin A.V., Grimmond S.M., Australian Pancreatic Cancer Genome Initiative (2014). Gemcitabine and CHK1 inhibition potentiate EGFR-directed radioimmunotherapy against pancreatic ductal adenocarcinoma. Clin. Cancer Res..

