# Supplementary material for: EphA3 Pay-Loaded Antibody Therapeutics for the Treatment of Glioblastoma
Source: Cancers (Basel). 2018 Dec 17;10(12):519. doi: 10.3390/cancers10120519 (PMC6316644; doi:10.3390/cancers10120519)
Supplement: Supplementary file 1 [file cancers-10-00519-s001.pdf]

Supplementary Materials: EphA3 Pay-Loaded Antibody Therapeutics for the Treatment of Glioblastoma

Carolin Offenhäuser, Fares Al-Ejeh, Simon Puttick, Kathleen S. Ensbey, Zara C. Bruce, Paul R. Jamieson, Fiona M. Smith, Brett W. Stringer, Benjamin Carrington, Adrian V. Fuchs, Craig A. Bell, Rosalind Jeffree, Stephen Rose, Kristofer J. Thurecht, Andrew W. Boyd and Bryan W. Day

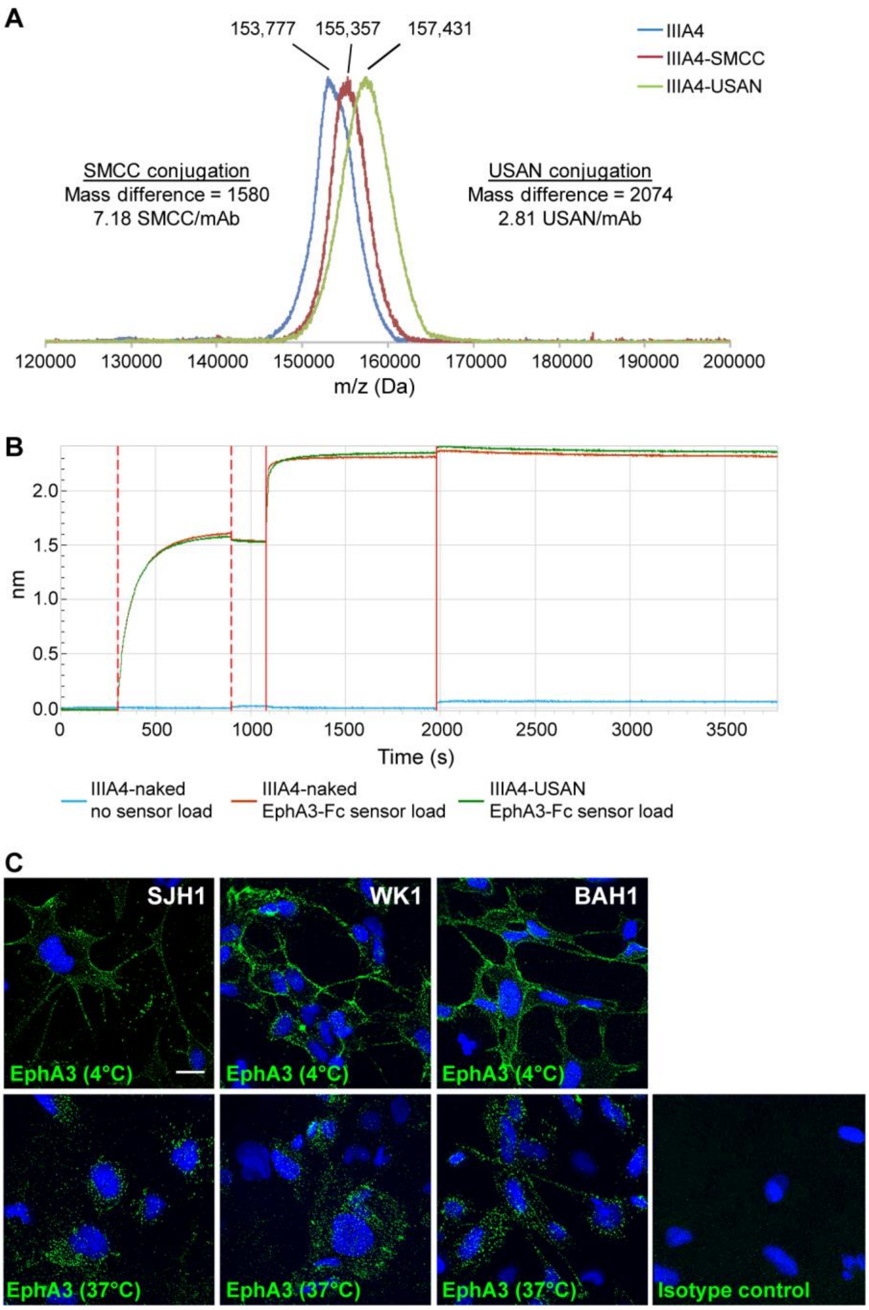

Figure S1. Validation of the EphA3-maytansine ADC IIIA4-USAN. (**A**) MALDI-ToF MS spectra of IIIA4 (blue trace), IIIA4-SMCC (red trace), and IIIA4-USAN (green trace). Observed shifts of the peak molecular weights were used to determine conjugation of both SMCC and USAN; the drug-antibody ratio (DAR) was calculated to be 2.81 USAN/mAb. (**B**) Binding kinetics of unconjugated

IIIA4 (IIIA4-naked) and IIIA4 conjugated to maytansine (IIIA4-USAN) using Bio-Layer Interferometry (BLI) technology. Biotinylated EphA3-Fc was immobilized onto streptavidin biosensors. Real-time binding charts were then acquired with the Octet® system to compare the binding kinetics of IIIA4-naked and IIIA-USAN (30 μg/mL) to the biosensors. (**C**) Representative immunofluorescence images of EphA3-labelling with IIIA4 mAbs (shown in green) on the cell surface (top panel) and after antibody internalization (bottom panel) in the patient-derived GBM cell line WK1, SJH1 and BAH1. An IgG1 antibody was used as an isotype control. Scale bar represents 10 μm.

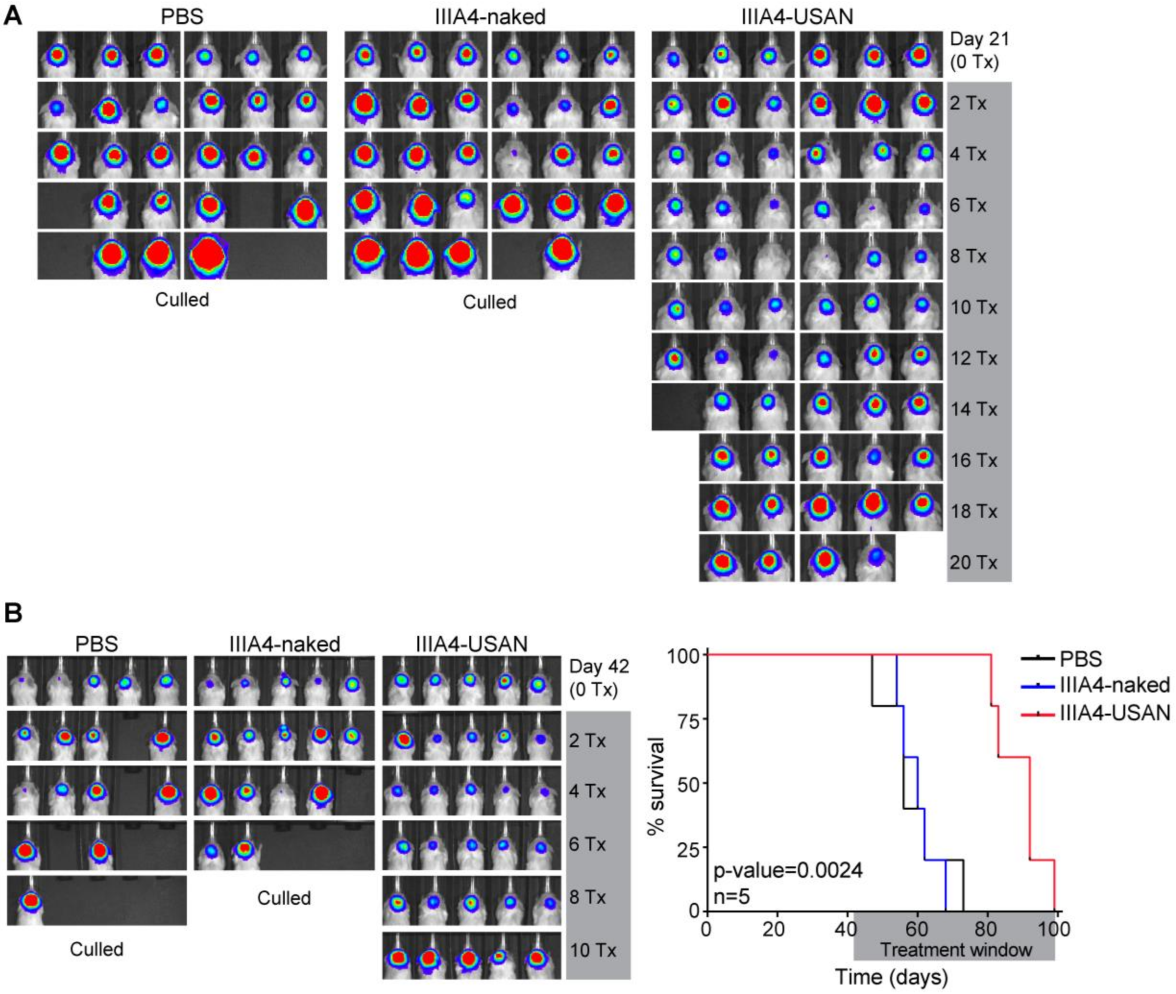

	PBS	IIIA4 naked	IIIA4 USAN
Median survival	56	60	92

Figure S2. In vivo efficacy of the EphA3-maytansine ADC IIIA4-USAN. (**A**) Complete imaging data for the experiment shown in Figure 4A. Luciferase-expressing U251 cells were engrafted intracranially. Starting from day 21, animals received twice weekly IV injections of IIIA4-USAN (10 mg/kg), IIIA4-naked (10 mg/kg) or PBS vehicle control. (**B**) Luciferase-expressing U251 cells were injected intracranially. On day 42, mice were randomized and tumour-matched based on bioluminescence imaging. Mice subsequently received twice weekly intravenous injections of 10 mg/kg IIIA4-USAN, IIIA4-naked or PBS vehicle control. Intravital bioluminescence imaging of luciferase activity in tumour cells and Kaplan-Meier survival plot are shown.

© 2018 by the authors. Licensee MDPI, Basel, Switzerland. This article is an open access article distributed under the terms and conditions of the Creative Commons Attribution (CC BY) license (http://creativecommons.org/licenses/by/4.0/).
